# Rab32 and Rab38 genes in chordate pigmentation: an evolutionary perspective

**DOI:** 10.1186/s12862-016-0596-1

**Published:** 2016-01-27

**Authors:** Ugo Coppola, Giovanni Annona, Salvatore D’Aniello, Filomena Ristoratore

**Affiliations:** Department of Biology and Evolution of Marine Organisms, Stazione Zoologica Anton Dorhn, Villa Comunale, 80121 Napoli, Italy

**Keywords:** Pigmentation, Amphioxus, Zebrafish, Synteny, Phylogeny, Intron Conservation, Gene Duplication, Genome Duplication

## Abstract

**Background:**

The regulation of cellular membrane trafficking in all eukaryotes is a very complex mechanism, mostly regulated by the Rab family proteins. Among all membrane-enclosed organelles, melanosomes are the cellular site for synthesis, storage and transport of melanin granules, making them an excellent model for studies on organelle biogenesis and motility. Specific Rab proteins, as Rab32 and Rab38, have been shown to play a key role in melanosome biogenesis. We analysed the Rab32 and Rab38 genes in the teleost zebrafish and in the cephalochordate amphioxus, gaining insight on their evolutionary history following gene and genome duplications.

**Results:**

We studied the molecular evolution of Rab supergroup III in deuterostomes by phylogenetic reconstruction, intron and synteny conservation. We discovered a novel amino acid stretch, named FALK, shared by three related classes belonging to Rab supergroup III: Rab7L1, Rab32LO and Rab32/Rab38. Among these, we demonstrated that the Rab32LO class, already present in the last common eukaryotic ancestor, was lost in urochordates and vertebrates. Synteny shows that one zebrafish gene, *Rab38a*, which is expressed in pigmented cells, retained the linkage with tyrosinase, a protein essential for pigmentation. Moreover, the chromosomal linkage of *Rab32* or *Rab38* with a member of the glutamate receptor metabotropic (Grm) family has been retained in all analysed gnathostomes, suggesting a conserved microsynteny in the vertebrate ancestor. Expression patterns of *Rab32* and *Rab38* genes in zebrafish, and *Rab32/38* in amphioxus, indicate their involvement in development of pigmented cells and notochord.

**Conclusions:**

Phylogenetic, intron conservation and synteny analyses point towards an evolutionary scenario based on a duplication of a single invertebrate *Rab32/38* gene giving rise to vertebrate *Rab32* and *Rab38*. The expression patterns of *Rab38* paralogues highlight sub-functionalization event. Finally, the discovery of a chromosomal linkage between the *Rab32* or *Rab38* gene with a *Grm* opens new perspectives on possible conserved bystander gene regulation across the vertebrate evolution.

**Electronic supplementary material:**

The online version of this article (doi:10.1186/s12862-016-0596-1) contains supplementary material, which is available to authorized users.

## Background

Intracellular compartmentalization, via membrane-delimited organelles, is a fundamental feature of the eukaryotic cell and membrane trafficking between organelles became vital for these cells.

These mechanisms are typically regulated by Rab proteins, which form by far the biggest family among the small GTPases, with more than 60 members in humans [1]. These proteins play a crucial role in the regulation of cellular membrane trafficking in all eukaryotes [2]. This role is orchestrated mainly by the switching between the GTP/GDP-bound states of these proteins, controlled by the guanine nucleotide exchange factors (GEF) and GTPase activating proteins (GAP). Most Rab GTPases consist of 220 amino acids and are roughly 24 kDa [3]. Rab proteins possess some conserved domains: the P-loop, a well-known nucleotide binding motif, fundamental for the switching between GTP/GDP-bound states; Switch I and Switch II that are necessary for the binding of guanine nucleotides [4]. Each Rab has a distinct subcellular localization and regulates a specific transport step. Evolutionary studies suggest that twenty Rab proteins, divided into six supergroups, were already present in the last eukaryotic common ancestor (LECA) [5]. The number of Rabs in metazoans is extremely variable due to the occurrence of several gene gains and losses [5]. The genomes of all eukaryotes encode multiple members of the Rab family, from 10–20 in most unicellular eukaryotes [6–8] to over 60 genes in multicellular organisms [9, 10].

The high number of Rab expansions and secondary losses in several eukaryotic lineages suggest a complex evolutionary history of this family [11]. Among all membrane-enclosed organelles, melanosomes represent an excellent model for studying the biogenesis and motility of these structures [12]. Melanosomes, lysosome-related organelles (LRO), are the cellular site for synthesis, storage and transport of melanin granules that provide colour to tissues and are involved in photoprotection. Melanosomes are present in mammalian skin melanocytes, in choroidal melanocytes, in retinal pigment epithelial (RPE) cells of the eye, and in melanophores of teleosts and amphibians [13]. Specific Rab proteins belonging to supergroup III, Rab32 and Rab38, play a key role in melanosome biogenesis [12]. This process has been well described in mammals where melanosome formation is conventionally divided into four steps. Rab32 and Rab38, together with effector proteins like AP-3, AP-1, and BLOC-2, mediate the transport of enzymes fundamental for pigmentation, such as metazoan tyrosinase (Tyr) and tyrosinase-related protein (Tyrp1) [14], from *trans*-Golgi network endosomes to maturing melanosomes (step II-III) [15].

It has been shown that a point mutation in the conserved GTP/GDP-interacting domain (P-loop) of the *Rab38* gene causes the *Chocolate* (*cht*) autosomal recessive mutation, which arose spontaneously in some mice strains [16]. The *cht* mice showed an oculocutaneous albinism phenotype and a weakly diluted coat colour. *Rab38* and the closely related *Rab32* work redundantly in melanocytes, as demonstrated by depletion of *Rab32* in *in vitro* cultured *cht* epidermal melanocytes, which severely impairs the transport of Tyr and Tyrp1 to melanosomes, resulting in severe hypopigmentation [12]. Mouse *Rab32* and *Rab38* are paralogues, sharing 67 % amino acid identity and are considered to have originated from the vertebrate whole genome duplication (WGD) that occurred before the Gnathostomata radiation [17–19].

Furthermore, it has been shown that the *Rab38* gene is mutated in ruby rats [20], a strain characterized by hypopigmentation and platelet storage pool deficiency related to Hermansky-Pudlak syndrome (HPS) [21]. This pathology in humans causes oculocutaneous albinism, easy bleedings, abnormal lysosomal ceroid lipofuscin and pulmonary fibrosis in 40–50 years-old patients [22]. Finally, frog (*Xenopus laevis*) melanophores are characterized by a strong expression of *Rab32*, suggesting an involvement in pigment formation [23].

Much less is known regarding invertebrate *Rab32/38* gene functioning: in the ascidian *Ciona intestinalis,* the closest living relative of vertebrates [24], the unique *Rab32/38* gene is expressed in four cells belonging to the pigment cell lineage [25]. Among these cells the otolith and ocellus, the sole pigmented sensory organs of ascidian larvae, will form [26]. Functional studies demonstrated that Rab32/38 is essential for proper pigmentation of otolith and ocellus pigmented cells [25].

In the fruit fly *Drosophila melanogaster Rab-RP1* (*Rab32*) is expressed in eye lysosomes and lipid droplets of adipose tissue [27], and its mutation causes eye hypopigmentation (*lightoid*) [28].

To gain insight into deuterostome evolution and the possible function of the *Rab32* and *Rab38* genes, we investigated the molecular evolution and spatio-temporal localization during development in two key animal models: the cephalochordate *Branchiostoma lanceolatum* and the teleost *Danio rerio*.

Cephalochordates represent an early-branching chordate group, their genomic, anatomical and morphological features, however, make them the best available stand-in for the chordate ancestor. Amphioxus has an unduplicated genome, but possesses representatives of all vertebrate gene families [29]. Amphioxus photoreceptive system is formed by 4 different structures: Joseph cells, the lamellar body, dorsal ocelli (Hesse cells) and the frontal eye [30]. Hesse cells are traditionally considered homologue of vertebrate eye photoreceptors, whereas the frontal eye pigment cells are thought to be homologue of vertebrate RPE cells, as demonstrated by *Mitf*, *Otx* and *Pax2/5/8* tissue-specific expression [31]*. Tyr*, *Tyrp1/2a* and *Tyrp1/2b* are co-expressed at the neurula stage in the neural tube where the first primary pigment spot will form [32].

Among vertebrates, the teleosts (32000 species) show an incredible variety of pigmentation patterns, decisive for their complex behaviour. Intriguingly, the skin pigmentation pattern of teleosts is not only due to the black/brown melanocytes, yellow/red xanthophores and reflecting iridophores, common to all vertebrates, but also to whitish leucophores and blue cyanophores that are lineage-specific novelties [33]. Teleosts are able to adapt perfectly to highly different environments, through the α-melanophore-stimulating hormone (α-MSH) secreted by the pituitary, which is influenced by neurotransmitters such as norepinephrine [34].

Teleost genomes have undergone a third round of whole genome duplication that probably provided the raw genetic material for the teleost radiation [35–37]. This event occurred only in the teleostean lineage, amongst actynopterigians [38], and it was therefore named “teleost-specific genome duplication” (TSGD) [39]. It has been suggested that a strong relationship exists between these genome duplications and the extraordinary pigmentation variety in teleosts: in fact teleost genomes show a dramatically wide repertoire of genes involved in pigmentation, produced both by TSGD and small-scale gene duplications [33]. Many pigmentation genes in teleosts gained other roles, distributing ancestral functions to duplicated genes (sub-functionalization) or acquiring new one after the split (neo-functionalization) [40].

In this paper, we studied all the *Rab32 and Rab38* genes present in the genomes of the amphioxus *B. lanceolatum* and the zebrafish *D. rerio* to shed light on the evolution of this subfamily of deuterostomes and gain insight into their role during chordate development.

## Results

### Evolutionary history of Rab32 and Rab38 in Deuterostomes

To study the evolutionary history of the Rab32 and Rab38 we performed a phylogenetic reconstruction starting from a database of manually curated supergroup Rab III sequences (see Additional files 1 and 2). We included sequences from vertebrates (*Petromyzon marinus*, *Callorinchus milii, Lepisosteus oculatus, Latimeria chalumnae, D. rerio*, *Xenopus tropicalis, Anolis carolinensis, Gallus gallus, Mus musculus,* and *Homo sapiens*), urochordates (*Ciona intestinalis*), cephalochordates (*B. lanceolatum*), hemichordates (*Saccoglossus kowalevskii*) and echinoderms (*Strongylocentrotus purpuratus*). Since Rabs are very similar to other small GTPase proteins, we only included sequences giving the higher Blast scores with known Rabs.

**Fig. 1:**
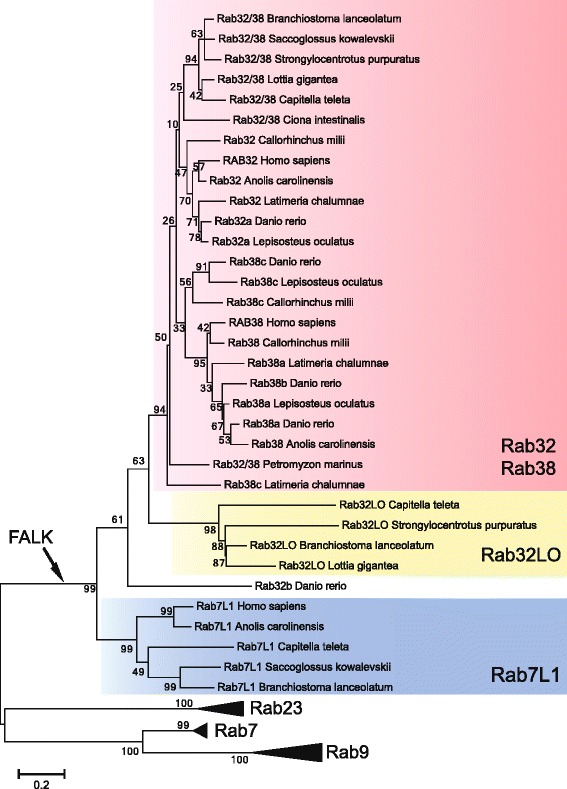
Evolution of the Rab32 and Rab38 subfamily in deuterostomes. Numbers at the branches indicate replicates obtained using the Maximum Likelihood estimation method. 247 sites were used for tree inference. Colored boxes highlight three classes of proteins: Rab7L1 present in invertebrates and vertebrates (*blue box*), Rab32LO present in protostomes, echinoderms and cephalochordates (*yellow box*), the Rab32 and Rab38 cluster present in all the deuterostomes (*red box*). Black arrow indicates the acquisition of FALK amino acid stretch at the stem of Rab7L1, Rab32LO, Rab32 and Rab38 evolution. Triangles indicate monophyletic clades belonging to Rab supergroup III (Rab7, Rab9, Rab23)

To obtain the best phylogenetic resolution we included representative members of Rab supergroup III: Rab32, Rab38, Rab7, Rab7L1, Rab9, Rab23 (see Additional file 2). Regarding Rab32 and Rab38, our analysis allowed to identify two genes in amphioxus and five in zebrafish genomes. Phylogenetic analysis revealed four strongly supported clades: Rab7, Rab9, Rab23 and a clade formed by Rab7L1, Rab32LO, Rab32 and Rab38. Among these, Rab7, Rab9 and Rab23 represent phylogenetically robust monophyletic groups (Fig. 1), while the Rab7L1 (blue box) results as sister group of Rab32LO (yellow box) and Rab32/Rab38 (red box).

Rab32LOs, previously named Rab32B [11], were described as already present in choanoflagellates (*Capsaspora owczarzaki)*, sponges (*Amphimedon queenslandica)* and cnidarians (*Nematostella vectensis)*, and therefore considered as LECA gene*.* Moreover, we found Rab32LO in protostomes (mollusks and platyhelminthes) and in deuterostomes (such as sea urchins and amphioxus). Strikingly, our study highlighted its loss in Olfactores (urochordates plus vertebrates) [24] prompting us to name it Rab32LO (Lost in Olfactores).

Unfortunately, our phylogenetic survey did not clarify the relationships among the vertebrate Rab32 and Rab38, and invertebrate Rab32/38 genes (Fig. 1 red box) due to the high degree of sequence similarity. The phylogenetic tree (Fig. 1) shows that amphioxus possesses a Rab32LO and a unique Rab32/38 member. Zebrafish presents three Rab38 (Rab38a, Rab38b and Rab38c), and two Rab32 genes (Rab32a and Rab32b). Strikingly, the position of Rab32b in the tree is inconsistent with species phylogeny, probably due to its fast evolutionary rate (Fig. 1). A vertebrate-specific phylogenetic analysis was performed to elucidate the relationship existing between Rab32 and Rab38 proteins in this group (see Additional files 3 and 4).

**Fig. 2:**
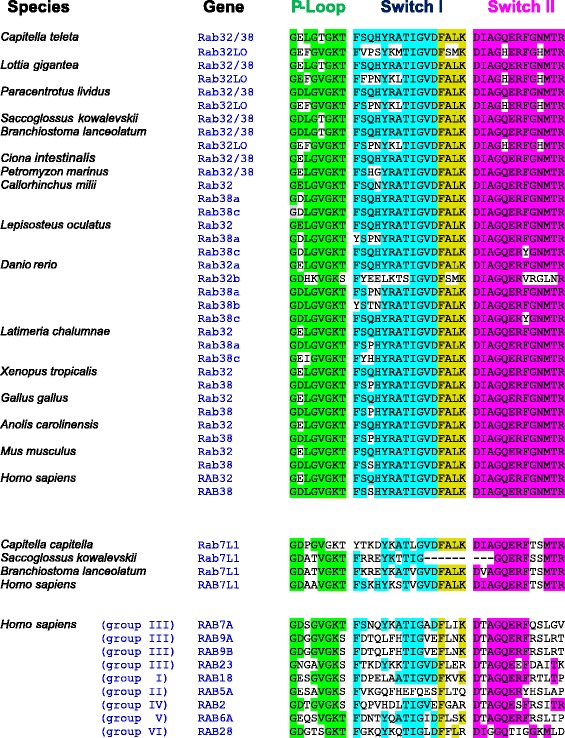
Alignment of Rab supergroup III domains. The alignment shows the amino acid conservation of three Rab domains: the P-loop, involved in trafficking (*green*), the Switch I (*turquoise*) and the Switch II (*magenta*) important for supporting the binding with other molecules. Downstream of Switch I, four ultra-conserved amino acids are highlighted, named FALK (*yellow*). The white background indicates changes in amino acid composition during evolution. At the bottom, human representatives of other Rab supergroups are reported to show the absence of FALK

With the aim to distinguish between Rab32 and Rab38 proteins, we compared the three conserved Rab domains across several deuterostome species, using the human RAB6A as reference [4]: the P-loop (green) located at amino acids 20–27, the Switch I (turquoise) at amino acid positions 38–49 and the Switch II (magenta) at amino acids 69–81 (Fig. 2). To better understand the conservation of protein domains, we also added all human proteins of Rab supergroup III and one representative of other five Rab supergroups. The alignment shows a high degree of conservation for three known domains in deuterostomes. The general core sequence of the P-loop is GxxxxGKT(S) in all subfamilies. We found that the 2^nd^ residue is diagnostic for Rab classification in vertebrates (GExxxGKT for Rab32s or GDxxxGKT for Rab38s). Switch I (consensus FSxxYxxTIGVD) and Switch II (consensus DIAGxERFGxMTR) are highly conserved across deuterostomes, showing sequence divergence only in zebrafish Rab32b. Moreover, we identified for the first time the existence of an ultra-conserved stretch of four amino acids (FALK) located at the end of Switch I that is present only in Rab32, Rab38, Rab32LO and Rab7L1 subfamily members (black arrow in Fig. 1, yellow in Fig. 2) among Rab supergroup III. The presence of two conserved Histidine residues exclusively in Rab32LO Switch II (Fig. 2) confirms the fact that Rab32LO is a distinct class within supergroup III, supporting our phylogenetic data. In order to clarify the poorly resolved relationship among invertebrate Rab32/38 and vertebrate Rab32 and Rab38 we searched for an evolutionary signature of conserved introns, analysing the gene structure of human *Rab32* and *Rab38* in comparison with *Branchiostoma*, *Ciona* and *Lottia Rab32/38* genes. Strikingly, we found two introns that show conserved position and phase, suggesting a common ancestral origin (see Additional file 5).

### *Rab32* and *Rab38* synteny conservation

**Fig. 3:**
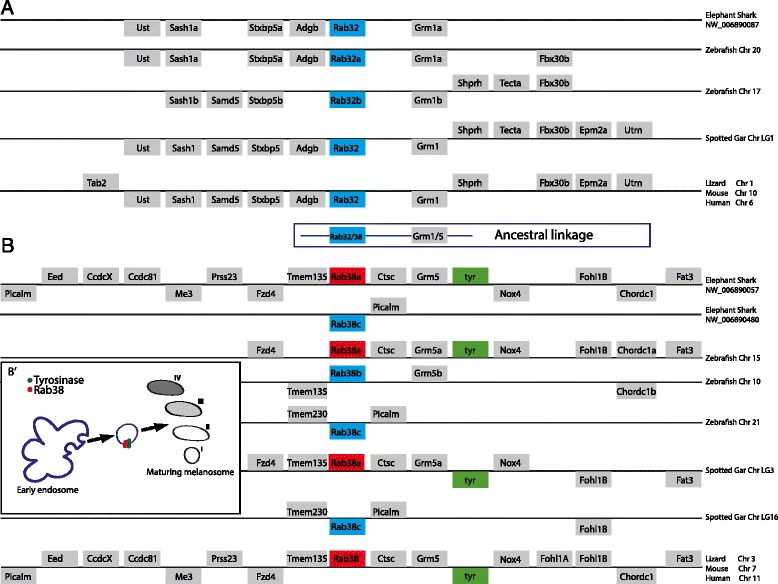
*Rab32* and *Rab38* synteny conservation in gnathostomes. The *Rab32* (**a**) and *Rab38* (**b**) loci harbour several genes (*grey boxes*) conserved across evolution; in blue boxes Rab genes we studied, in red boxes Rab38 genes that are physically linked to tyrosinase (*green*). During the gnathostome evolution, the *Rab32* is always linked to Grm1, while Rab38 is linked to Grm5. The scheme 3**b** ^**’**^ shows the functional relationship between Rab38 and Tyr during mammalian melanosome biogenesis (adapted from [55]). The position of the genes above or below the chromosome (*horizontal line*) indicates their transcriptional orientation on positive or negative strand, respectively

To further improve knowledge on the evolutionary history of the *Rab32* and *Rab38* subfamilies, we analysed their chromosomal neighbourhood genes in available deuterostome genomes (Fig. 3). We searched in genomes of invertebrates (*S. kowalevskii, S. purpuratus, B. floridae, C. intestinalis*) and, among vertebrates, we selected the lamprey *P. marinus* for the agnathans, the elephant shark *C. milii* [41] and spotted gar *L. oculatus* as representatives of non-teleost fish [42], *D. rerio* for teleosts, the lizard *A. carolinensis* in the Sauropsida clade, and *H. sapiens* and *M. musculus* among mammals. We did not find chromosomal conservation in lamprey. On the other hand, we discovered a high degree of synteny conservation within both gnathostome *Rab32s* loci (Fig. 3a) and *Rab38s* loci (Fig. 3b). Nevertheless, the genes flanking *Rab32* are different from those surrounding *Rab38*. To demonstrate the orthology of syntenic genes we performed *ad hoc* phylogenies of *Tab*, *Nox*, *Fzd* and *Stxbp* genes (see Additional file 6).

The existence of a chromosomal region conserved between *H. sapiens* and some teleosts, containing *Rab38*, *Grm5* and *Tyr* genes has been already described in a comparative study [33]. Our syntenic survey across the vertebrates extended the conservation to the holocephalan elephant shark, the non-teleost actinopterygian spotted gar, the anole lizard and mice. Moreover, we also showed that *Rab32* genes are always linked to a *Grm* family member (*Grm1*) (Fig. 3). Collectively, our data suggest an ancestral chromosomal linkage between a *Rab32/38* and *Grm1/5* gene, already present in the vertebrate’s common ancestor (Fig. 3). We carried out the phylogeny of the *Grm* family to demonstrate the orthology among vertebrate *Grm1* genes and *Grm5* genes, respectively (see Additional file 7), further supporting our hypothesis of an ancient chromosomal linkage. In more detail, we found one *Rab32* always coupled with the melanoma oncogene *Grm1* [43, 44], while one *Rab38* is always close to *Grm5*, a gene playing a fundamental role in many human disorders such as schizophrenia [45] and autism [46].

Among zebrafish *Rab38* paralogues we found a higher degree of syntenic conservation in the *Rab38a* locus (number of genes and organization) than in the *Rab38b* and *Rab38c* loci (Fig. 3b). For instance, we found that only the zebrafish *Rab38a* maintained the linkage with the *Tyr* gene, as is the case in the unique tetrapod *Rab38* paralogue. Similarly, in elephant shark and spotted gar this linkage is retained in only one of the two *Rab38* paralogons (Fig. 3b).

Additionally, in tetrapod *Fohl1A* and *Fohl1B*, and the Coiled-coil domain containing proteins (*Ccdc*), belonging to a big family whose role is completely unknown, are duplicated on the same chromosomal stretch (Fig. 3b). The mammalian *Rab38* loci show an interesting peculiarity, they present several insertions of receptors belonging to the same family derived from tandem gene duplication events (see Additional file 8A). On mouse chromosome 7, beside the two *Fohl1s*, there are fifteen *Vomeronasal 2 Receptors* (*Vmn2r*), known for their crucial role in mouse ultrasensitive chemodetection [47] and fourteen *Olfactory Receptors* (*OR*) [48], belonging to a very large family in mice, explaining their extraordinary odour sensitivity and ability to discriminate scents [47, 48]. Interestingly, in the orthologous position, the human chromosome 11 harbours seven tripartite motif proteins (*TRIM*), which are involved in a plethora of biological processes, in particular the immune response [49] (see Additional file 8A).

On the other hand, we did not find any synteny conservation in Rab loci of invertebrate deuterostomes. The unique trace of microsynteny was found in the sea urchin and amphioxus gene scaffolds, between *Rab32LO* and *Tim9*, an evolutionarily conserved transporter involved in metabolite import by mammalian mitochondria [50] (see Additional file 8B).

### *Rab32* and *Rab38* gene expression patterns in amphioxus and zebrafish

**Fig. 4:**
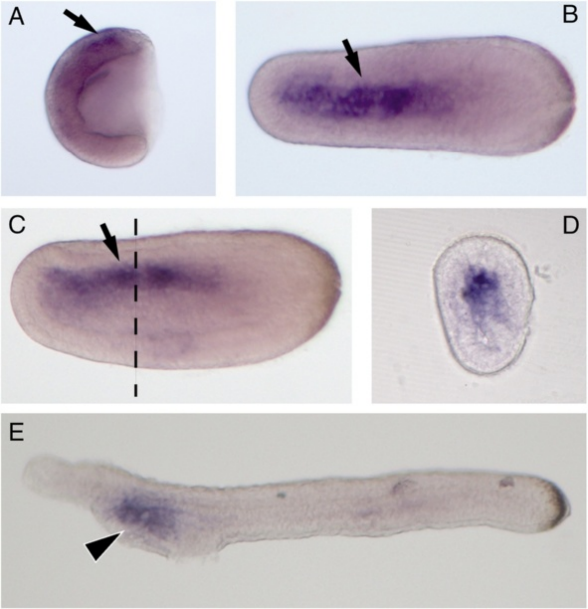
*Rab32/38* expression pattern during amphioxus embryogenesis. *Rab32/38* has been observed in notochord presumptive territories at the gastrula stage (**a**, *black arrow*), while later in development at the neurula stage it is expressed in notochord, mainly in the rostral part (**b-c**, *black arrow*). **d** is a vibratome section (15 μm) of the neurula specimen showed in **b-c**, at the level of the vertical dashed line. At the pre-mouth larval stage, *Rab32/38* expression in the notochord turns off while a positive signal appears in the pharynx region (**e**, *arrowhead*)

To investigate the possible role of Rab genes in the amphioxus *B. lanceolatum*, we cloned the *Rab32LO* and *Rab32/38* genes and studied their expression pattern by whole mount *in situ* hybridization (WISH) in embryos at different developmental stages (Fig. 4). Unfortunately, *Rab32LO* was not detectable by *in situ* hybridization at any analysed stage, confirmed by the low levels of expression detected by real-time RT-PCR experiments (see Additional file 9). *Rab32/38* was first observed at the gastrula stage in presumptive notochord territories (Fig. 4a), and later during development, at the neurula stage, it appears clearly in the notochord, from the most rostral part along the length of the embryo without reaching the caudal part (Fig. 4b, c). The *Rab32/38* expression in the notochord has been confirmed by the transverse section (Fig. 4d). At the pre-mouth larval stage of development, the *Rab32/38* expression in the notochord turns off and a novel territory of expression is visible in the pharynx region (Fig. 4e).

**Fig. 5:**
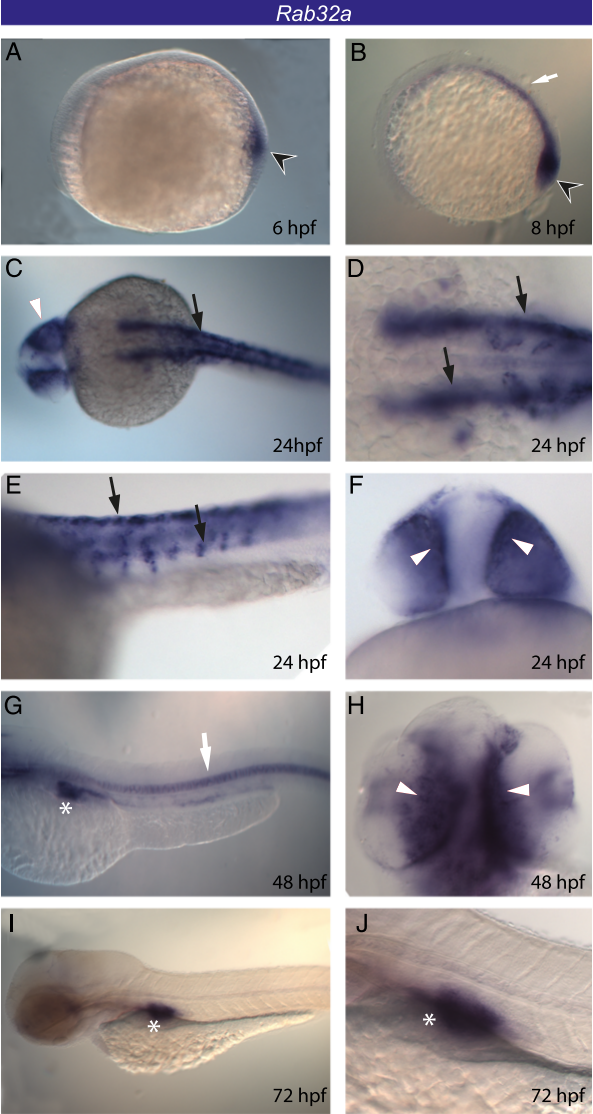
*Rab32a* expression pattern during zebrafish embryogenesis. *Rab32a* has been observed in the presumptive posterior axial mesoderm (*arrowhead*) at 6 hpf (**a**) and in the developing notochord (*white arrow*) and Kupffer’s vesicle (*arrowhead*) at 8 hpf (**b**). At 24 hpf, it is present in RPE (*white arrowhead*), notochord and migrating neural crest cells (black arrows) (**c-f**). At 48 hpf the signal disappears in neural crest cells, but persists in RPE and notochord (*white arrows*) (**g-h**) and appears in the swim bladder (**g**, *white asterisk*). The expression in the swim bladder persists at 72 hpf larvae (**i-j**, *white asterisk*)

The same approach was used to investigate the expression pattern of *Rab32* and *Rab38* during zebrafish embryogenesis. It was impossible to amplify *Rab32b* from embryonic and adult tissues (brain and eye), suggesting that this gene is either expressed at very low levels or only under specific environmental conditions. *Rab32a* expression has been detected in the presumptive posterior axial mesoderm starting from 6 h post-fertlization (hpf) (shield stage), as it was already described by Thisse and collaborators [51] (Fig. 5a). During embryo elongation (tail bud stage), the *Rab32a* positive cells were localized near the animal pole and form a longitudinal band in the dorsal midline, the developing notochord that includes the tail bud region (Fig. 5b). Moreover, at this stage, a signal is detectable in the Kupffer’s vesicle (Fig. 5b), a small but distinctive epithelial sac, situated posteriorly near the yolk [52]. During segmentation (24 hpf), the expression of *Rab32a* becomes weaker in the notochord (Fig. 5c), while it starts to be expressed in the eyes, as well as in cells that appear to be migrating melanoblasts according to their position (Fig. 5c-f). Starting from the long-pec stage (48 hpf), the expression in migrating melanoblasts disappears and is retained only in the retinal pigmented epithelium of the eye and in the notochord (Fig. 5g, h). At this stage, a strong signal becomes visible (Fig. 5g) in the swim bladder, the organ that adult teleosts use for buoyancy and breathing, and persists up to the protruding-mouth larval stage (72 hpf; Fig. 5i, j).

**Fig. 6:**
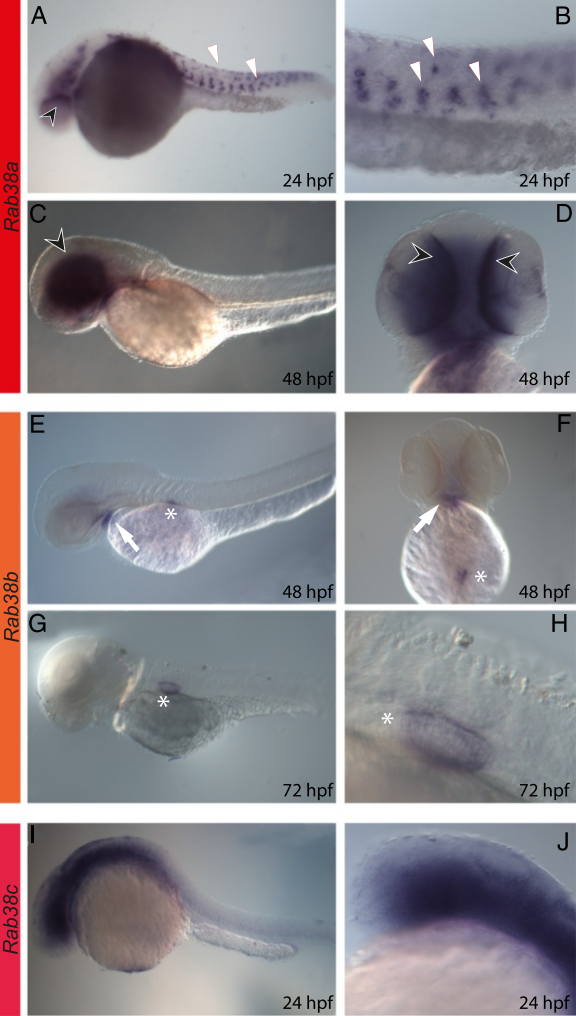
*Rab38s* expression pattern during zebrafish embryogenesis. *Rab38a* is expressed across the pharyngula embryonic period (24–48 hpf) (**a-d**): at 24 hpf there is a faint signal in RPE (*arrowhead*) and a strong one in migrating melanoblasts (**a**, **b**, *white arrowhead*) and a low level of expression is detectable in the mid-ventral region of brain (**a**); at 48 hpf the expression is visible only in the RPE (*black arrowhead*) (**c**, **d**). *Rab38b* is expressed only at late developmental stages (**e-h**): at 48 hpf in a small region of the pharyngeal arches (*white arrow*) and in the developing swim bladder (**e**, **f**, *white asterisks*), while at 72 hpf only in swim bladder (**g**, **h**, *white asterisks*). The gene *Rab38c* gene is strongly expressed in the head region (**i** and **j** for 24 hpf larvae). Lateral view in all images (anterior is on the left) except **d** and **f** that are ventral (anterior on the top)

Regarding *Rab38* paralogues, *Rab38a* is expressed across the pharyngula embryonic period (24–48 hpf) (Fig. 6a-d). Embryos at 24 hpf show a faint signal in RPE and a strong one in migrating neural crest cells, presumably migrating melanoblasts (Fig. 6a, b). At this stage, a low level of expression is detectable in the mid-ventral region of brain as well (Fig. 6a). Later in development (48 hpf) expression is only observed in the RPE (Fig. 6c, d). *Rab38b* is expressed only at late developmental stages (Fig. 6e-h); at 48 hpf it is expressed in a small region of the pharyngeal arch and in the developing swim bladder (Fig. 6e, f), while at 72 hpf only the signal in the swim bladder persists (Fig. 6g, h). The gene *Rab38c* is expressed from 6 to 72 hpf with a strong signal in the head (as shown in Fig. 6i, j for 24 hpf).

## Discussion

### Rab32 and Rab38 toolkit in animal evolution

Our phylogeny evidences three distinct branches: Rab7L1, Rab32LO and Rab32/Rab38, which share the highly conserved FALK stretch downstream of the Switch I domain and emerged after splitting from Rab23, Rab7 and Rab9 groups (Fig. 1). Although the FALK function is still unknown, its high degree of sequence conservation in all analysed proteins suggests that it could be responsible for important functional properties (Fig. 2).

It has been suggested that the Rab32/38, already described as Rab32A and Rab32LO, previously named Rab32B, present in the Last Eukaryotic Common Ancestor were already implicated in late endosomal or lysosomal trafficking [11]. Rab32LO is present in unicellular eukaryotes to chordates [11], and here we showed its loss in the lineage of Olfactores. This event could be related to the loss of ancestral functions during chordate evolution, although the biological functions of these proteins should be further investigated in the future.

Detailed analysis of the conserved Rab functional domains allowed to identify few diagnostic residues of vertebrate Rab32 and Rab38 (Fig. 2). Similarly, two Histidines are distinctive residues of the Rab32LO Switch II region. Our analyses suggest that the phylogenetic signal is weak for the heterogeneous evolutionary rate of this group of proteins, leaving the issue of vertebrate Rab32 and Rab38 origin unresolved. A possible hypothesis is that vertebrate *Rab32* and *Rab38* arose from a prevertebrate *Rab32/38* through a vertebrate genome duplication. The study of intron conservation, considered a diagnostic tool for tracing back the evolutionary history of genes [53], showed the existence of a conserved intron code supporting a common origin for all the subfamily members (see Additional file 5). Furthermore, the parallel evolutionary history of *Rab32/38* and *Grm1/5* genes is consistent with our hypothesis of a duplication event in vertebrates. In fact, in invertebrates, similarly to the *Rab32/38*, a single pre-duplicative *Grm1/5* exists as demonstrated by the dedicated phylogeny (see Additional file 7). Interestingly, the chromosomal proximity of *Rab32* to *Grm1* and *Rab38* to *Grm5* indicates a duplication of an ancestral chromosomal region comprising *Rab32/38* and *Grm1/5* (Fig. 3). The retained microsynteny that we found from elephant shark to humans hints at a functional correlation between these two gene duplets. It would be challenging to find out whether the integrity of these linkages is necessary for their functioning. Collectively our phylogeny, intron and synteny conservation analyses suggest that *Rab32* and *Rab38* derived from a vertebrate genome duplication, therefore they are both ohnologs of the pre-vertebrate *Rab32/38* gene.

We found a very high degree of chromosomal conservation among vertebrates (Fig. 3). In particular the high similarity between *C. milii Rab38a* and tetrapod *Rab38* loci confirms that cartilaginous fishes mimic the tetrapod genomic organization better than teleosts [54]. Surprisingly, a sharp lack of synteny involving only one side of the *Rab38a* of spotted gar (LG16) and zebrafish (chromosome 15) loci suggests a rearrangement event in the Actinopterygii (Fig. 3b).

Vertebrates have been affected by several rounds of genome duplication that have increased the repertoire of pigmentation genes mainly in teleosts [40]. The present survey highlights the expansion of the *Rab32* and *Rab38* driven by a mixture of gene and genome duplications that allowed the emergence of novel functions [34]. In zebrafish the *Rab32* and *Rab38* reached a total of five members through the TSGD, plus single gene duplication in the *Rab38* lineage.

The role of TSGD in the evolution of teleost pigmentation diversity is well-known resulting in 30 % more pigmentation genes than in tetrapods. In fact, other genes involved in pigmentation are also duplicated such as *Mitf*, *Kitl*, *Tyr* and *Tyrp* [33, 40] explaining the extremely diversified body colour patterning of teleosts [40].

Intriguingly, we noticed in gnathostomes the existence of a specific linkage among two well-known players of the pigmentation process; the *Tyr* and *Rab38*, retained in all analysed tetrapods and in the elephant shark, spotted gar and zebrafish *Rab38a* paralogons (Fig. 3b). It has been demonstrated that the delivery of Tyr, Tyrp1 and Tyrp2 to the maturing melanosomes is required to initiate pigmentation. Rab32 and Rab38 have been shown to mediate the transport of Tyr and Tyrp1 by interacting with the ubiquitous trafficking machinery [55] (Fig. 3b^’^). Taking into consideration that *Rab38a* is the sole *Rab38* member to be expressed in zebrafish pigment-producing cells and its chromosomal vicinity to *Tyr*, we hypothesize a bystander gene regulation modality during vertebrate melanosomes biogenesis by means of a locus control region, as previously demonstrated for developmental genes [56].

### Expression profile in zebrafish and amphioxus

In mammals, melanocytes, platelets and mast cells, rich in LROs, exhibit a high level of *Rab32* [57], whereas *Rab38* is restricted to melanocytes and lung epithelial cells [58, 59]. In zebrafish, we have identified two *Rab32* and three *Rab38* genes (Fig. 1). *Rab32a* is expressed in the embryonic pigmentary lineage, including both melanocytes and RPE, as described in other vertebrates. Moreover, it is expressed during notochord development, a feature that seems to be specific of zebrafish ([51] and present study). Recently it has been proposed that Rab32a function is required for correct formation of the notochord vacuoles in zebrafish [60]. Finally, *Rab32a* is expressed in the natatory vesicle of zebrafish (Fig. 5), also known as the swim bladder, an organ whose relationship with the tetrapod lung is still debated [61]. Considering that invertebrate Rab32/38 has an important role in melanogenesis, as demonstrated by a functional study in *C. intestinalis* [25], and that this role is maintained in vertebrate Rab32, we can assume Rab32 function in melanogenesis as ancestral. In zebrafish this gene has acquired new roles in the formation of other lysosome related organelles.

Zebrafish shows a varied scenario of *Rab32s* and *Rab38s* expressions with a high spatio-temporal diversification due to genomic events that occurred during the evolution of vertebrates. The three zebrafish *Rab38s* are expressed in distinct embryonic territories (Fig. 6), including pigmented cells, swim bladder and nervous system, unravelling a functional paralogous diversification. Distinct types of duplications have permitted the functional specialization in pigmentation of one of the paralogues [40]. The TSGD provided further raw genetic material for the evolution of teleost pigmentation patterns [33, 40]. The expression data of the three zebrafish *Rab38s* are an example of ancestral function distribution among duplicates (sub-functionalization).

Finally, the amphioxus *Rab32/38* expression territories in the notochord and pharynx (Fig. 4b, c, d) are consistent with transient zebrafish *Rab32a* expression in the notochord and *Rab38b* in pharyngeal arches. This findings suggest possible ancestral roles of the prechordate Rab32/38, which seem to be lost in tetrapods.

## Conclusions

Our study focused on genes belonging to the Rab family known to be involved in melanosome formation in mammals: Rab32 and Rab38. Phylogenetic, intron conservation and synteny analyses of these genes in deuterostomes point towards an evolutionary scenario based on a duplication of a single invertebrate Rab32/38 gene giving rise to vertebrate ohnologs Rab32 and Rab38. Additional duplicates arose in bony fishes by teleost-specific genome duplication and expression pattern of Rab38 paralogues in zebrafish evokes sub-functionalization event. Finally, the discovery of a chromosomal linkage between the Rab32 or Rab38 gene with a Grm1 or Grm5 opens new perspectives on possible conserved bystander gene regulation across the vertebrate evolution. However, future studies are necessary to investigate the presence of shared enhancer and to test functionally its regulative properties.

## Methods

### Phylogenetic analysis

The sequences used for the evolutionary analysis were retrieved from the NCBI (http://www.ncbi.nlm.nih.gov) and Ensembl (http://www.ensembl.org/index.html) databases (see Additional files 1 and 2). *C. intestinalis* Rab32/38 was the initial query sequence used for tBlastn [62] in invertebrate genomes, and reciprocal blasts were performed on each genome. The proteins were aligned by ClustalW with default parameters [63]. The phylogenetic trees were computed employing the Maximum-Likelihood estimation (MLE) using MEGA6 with 1,000 replicates and the WAG + γ + I matrix [64]. The graphical representation of trees was created with Dendroscope [65]. The main Rab domains showed in Fig. 2 have been aligned manually. The synteny among deuterostome genomes was studied by manually mapping the genes on the scaffolds/chromosomes using available public resources.

### Cloning and riboprobe preparation

Amphioxus *Rab32/38* was cloned from a mix of embryonic stages from gastrula to 5 days-old larvae fixed in Trizol (EuroClone). Zebrafish *Rab32* and *Rab38* genes were cloned using the prediction available in the NCBI database: *Rab32a* (BC066502), *Rab38a* (XM_001342839.2), *Rab38b* (XM_003199354.1) and *Rab38c* (XM_685900.3). For all of them except the last one, we found expressed sequence tags (ESTs) in several developmental stages and body structures (see Additional file 10). The cDNA sequences were PCR-amplified using the following oligonucleotide pairs: BlRab32/38-F (CACAAACCTCACACCTTCCA) and BlRab32/38-R (TGGTTCATCTGTGCTCGTTC) for amphioxus *Rab32/38*, BlRab32LO-F (TCGGACAGCAGAAACAACAC) and BlRab32LO-R (CTGCTCAGCTTCAGGATGTG) for amphioxus *Rab32LO*, DrRab32a-F (GTTGCACAGAGTTGCCAAAA) and DrRab32a-R (GTGTCTGTCAACCCCTGGAT) for zebrafish *Rab32a*, DrRab38a-F (TGGGGAAAACCAGCATTATC) and DrRab38a-R (TGCTGCGGTGAAATAGTGTC) for zebrafish *Rab38a*, DrRab38b-F (CATGACGCGGGTTTATTACA) and DrRab38b-R (TGGGTCCTTATCGGTGACTT) for zebrafish *Rab38b*, and DrRab38c-F (GCATCTGTTCAAAGTTCTGG) and DrRab38c-R (TGACTTGGAACACGTCATGC) for zebrafish *Rab38c*. The amplified gene fragments were cloned with the TOPO-TA II Cloning Kit (Invitrogen). Correct cloning was confirmed by sequencing of both DNA strands. One μg of purified DNA was used for *in vitro* transcription of the DIG-labeled riboprobes, using SP6 and T7 RNA polymerases (Roche). The ribonucleic probes were purified using 4 M lithium chloride (LiCl) and stored at −80 °C until use. The transcripts abundance in amphioxus (*Branchiostoma lanceolatum*) was evaluated by real time RT-PCR (see Additional file 9).

### Amphioxus and zebrafish embryos

Adult amphioxus (*Branchiostoma lanceolatum*) were collected in the Gulf of Naples (Italy) and maintained in an open seawater circulation aquaculture under a 14 h light/10 h dark cycle. Animals were reared in tanks with 10 cm of sand from the collection site and fed daily with a mix of the following unicellular algae: *Dunaliella tertiolecta*, *Isochrysis galbana* and *Tetraselmis suecica*. Spawning was induced in the laboratory in late spring by applying a thermic shock, as reported in Fuentes *et al.* [66]. After *in vitro* fertilization, embryos were cultured in 0,22 μm filtered seawater at 18 °C in plastic petri dishes, and fixed at different developmental stages with 4 % paraformaldehyde (PFA) in MOPS buffer overnight at 4 °C, and then stored in 70 % ethanol at −20 °C.

Zebrafish (*Danio rerio*) embryos up to 4 dpf were obtained from natural spawning of wild-type animals and fixed overnight in 4 % PFA dissolved in phosphate buffered saline (PBS), then washed in PBS and kept in methanol at −20 °C.

### Animal ethics

The protocols for handling of zebrafish and experiments involving not feeding larvae were approved by the ethical committee of the Stazione Zoologica Anton Dohrn of Napoli, Italy (Animal Welfare Body).

### Whole-mount *in situ* hybridization

Whole-mount *in situ* hybridization (WISH) in amphioxus was performed as described in Irimia *et al*. [67]. Briefly, after embryo re-hydration, digestion with Proteinase K (5 μg/ml) was performed to facilitate the riboprobe penetration; the reaction was stopped by adding 4 μl of 10 % glycine and then washed with 2 mg/ml glycine in a phosphate buffered saline solution containing Tween20 0.1 % (PBT). The embryos were refixed in PBT containing 4 % PFA for 1 h at RT and then washed in 0.1 M triethanolamine with acetic anhydride to bleach the natural pigments of the embryos. Embryos were washed with PBT several times and hybridized ON at 65 °C in DEPC-H_2_O hybridization buffer (50 % deionized formamide; 100 μg/ml Heparin; 5× SSC; 0.1 % Tween20; 5 mM EDTA; 1× Denhardt’s 1 mg/ml; 50 mg/ml yeast RNA).

In zebrafish, whole-mount *in situ* hybridization (WISH) was performed as described by Thisse *et al.* [68]. Briefly, after embryo re-hydration, digestion with Proteinase K (10 μg/ml) was performed to permeabilize the embryonic tissues, and the reaction was stopped by fast washes in PBT. The embryos were refixed in 4 % PFA in PBT for 1 h at RT and washed with PBT many times. They were hybridized ON at 65 °C in hybridization buffer in DEPC-H_2_O (50 % Formammide; 100 μg/ml Heparin; 1.3X SSC; 0.2 % Tween20; 5 mM EDTA pH 8,0; 50 μg/ml Yeast RNA; 0.5 % CHAPS).

Embryo image capturing was performed with a Zeiss Axio Imager M1.

## Additional files

Additional file 1:
**Accession number of sequences used in the evolutionary analysis.** (XLSX 19 kb) Additional file 2:
**Database of sequences used in phylogeny of Fig.** **1** **.** (TXT 14 kb) Additional file 3:
**Vertebrate Rab32 and Rab38 phylogeny.** (PDF 178 kb) Additional file 4:
**Database of sequences used in phylogeny of Additional file** **3** **.** (TXT 5 kb) Additional file 5:
**Intron conservation.** This survey inferred the existence of a conserved intron code in Rab32 and Rab38 subfamily retained after a vertebrate duplication. Switch I (partial) and Switch II domains are underlined. (DOCX 15 kb) Additional file 6:
**Phylogenies of Tab, Nox, Fzd, Stxbp.** (PDF 378 kb) Additional file 7:
**GRM phylogeny.** (PDF 282 kb) Additional file 8:
**Additional syntenic studies.** **A**. Human chromosome 11 and mouse chromosome 7: a focus on Rab38 gene loci with, respectively, a TRIM gene family expansion and an extraordinary clustering of olfactory genes: Vomeronasal 2 receptor (Vmn2r) and Olfactory Receptor (OR) in mouse; **B** Microsynteny of Rab32LO between echinoderms and cephalochordates. (PPTX 72.8 kb) Additional file 9:
**Gene expression profile during amphioxus development.** Rab32/38 and Rab32LO gene expression profiles by real time RT-PCR during *Branchiostoma lanceolatum* development. Both genes have been normalized using the ribosomal protein L32 (RPL32) expression using the same cDNA template. (DOCX 41.4 kb) Additional file 10:
**Zebrafish ESTs available in NCBI database.** (XLSX 10.5 kb)
