# Supplementary figures and images for: Rab32 and Rab38 genes in chordate pigmentation: an evolutionary perspective
Source: BMC Evol Biol. 2016 Jan 27;16:26. doi: 10.1186/s12862-016-0596-1 (PMC4728774; doi:10.1186/s12862-016-0596-1)

## Additional file 3: vertebrate Rab32 and Rab38 phylogeny

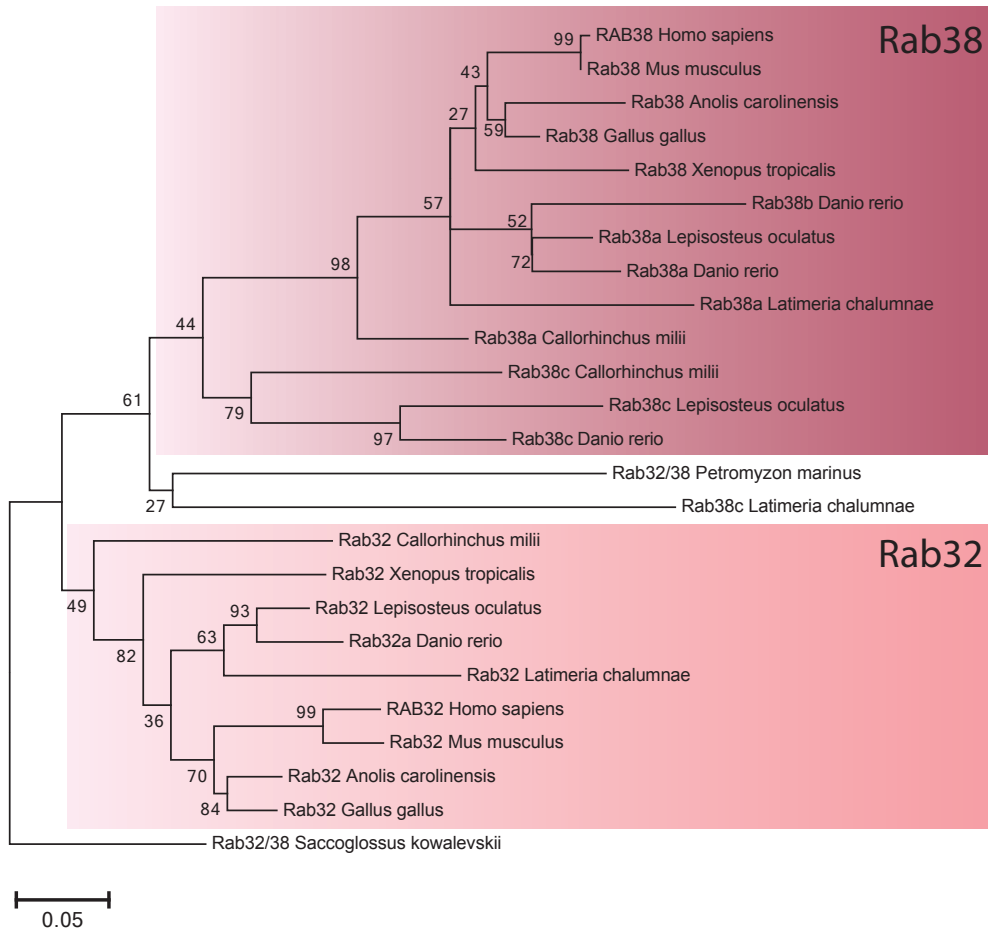

Supplement: Additional file 3: — Vertebrate Rab32 and Rab38 phylogeny. (PDF 178 kb) [file 12862_2016_596_MOESM3_ESM.pdf]

## Additional file 7: GRM phylogeny

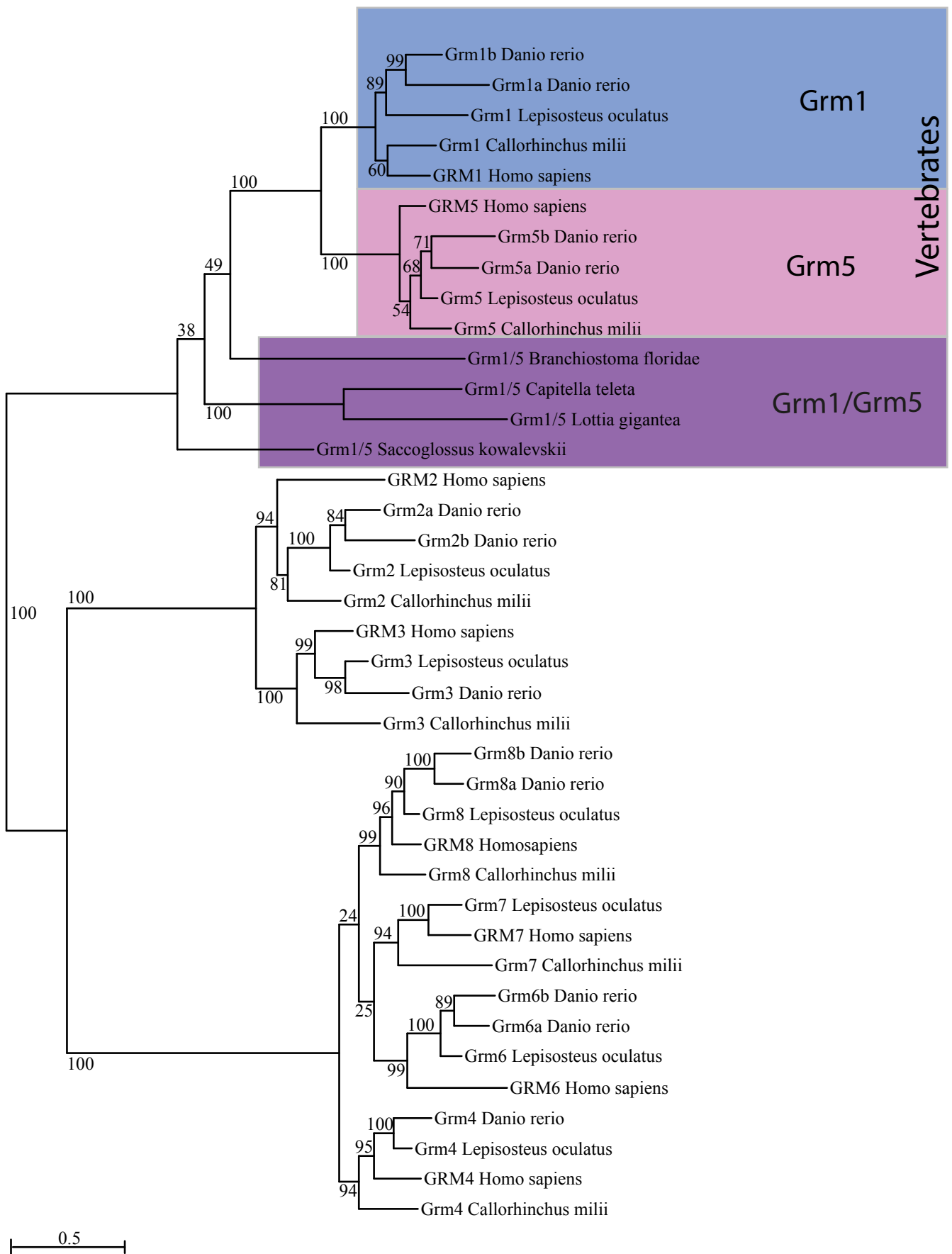

Supplement: Additional file 7: — GRM phylogeny. (PDF 282 kb) [file 12862_2016_596_MOESM7_ESM.pdf]
