# Supplementary material for: Rab32 and Rab38 genes in chordate pigmentation: an evolutionary perspective
Source: BMC Evol Biol. 2016 Jan 27;16:26. doi: 10.1186/s12862-016-0596-1 (PMC4728774; doi:10.1186/s12862-016-0596-1)
Supplement: Additional file 5: — Intron conservation. This survey inferred the existence of a conserved intron code in Rab32 and Rab38 subfamily retained after a vertebrate duplication. Switch I (partial) and Switch II domains are underlined. (DOCX 15 kb) [file 12862_2016_596_MOESM5_ESM.docx]

**Additional file 5: Intron conservation**

This survey inferred the existence of a conserved intron code in Rab32 and Rab38 subfamily retained after a vertebrate duplication. Switch I (partial) and Switch II domains are underlined.

Ancestral Intron

phase 1

Lottia Rab32/38 VDFALKVLNWDSDTLIRLQLWDIA 1 GQERFGNMTRVYYKEAVGAFVVFDVTRA

Amphioxus Rab32/38 VDFALKVINWDADTLIRLQLWDIA 1 GQERFGNMTRVYYKEAVGAFVVFDVTRA

Ciona Rab32/38 VDFALKVVHWDGETLIRLQLWDIA 1 GQERFGNMTRVYYREAVGAFIVFDATRA

Human Rab32 VDFALKVLNWDSRTLVRLQLWDIA 1 GQERFGNMTRVYYKEAVGAFVVFDISRS

Human Rab38 VDFALKVLHWDPETVVRLQLWDIA 1 GQERFGNMTRVYYREAMGAFIVFDVTRP

Ancestral Intron

phase 0

Lottia Rab32/38 STFDAVSKWKNDLDSKVQLPDGTPVPCVLLANK 0 CDQAKEGLVNNSAQMDEF

Amphioxus Rab32/38 STFEAVSKWKNDLDSKVQLPNGQSIPAVLLANK 0 CDQAKEGMVNSTSQMDEY

Ciona Rab32/38 STFEAVNKWKNDLDNKVTLPDGRNIPTVLLGNK 0 IDQMRGGLLSNKQQMDNY

Human Rab32 STFEAVLKWKSDLDSKVHLPNGSPIPAVLLANK 0 CDQNKDSSQ-SPSQVDQF

Human Rab38 ATFEAVAKWKNDLDSKLSLPNGKPVSVVLLANK 0 CDQGKDVLMNNGLKMDQF
