# Supplementary material for: Rab32 and Rab38 genes in chordate pigmentation: an evolutionary perspective
Source: BMC Evol Biol. 2016 Jan 27;16:26. doi: 10.1186/s12862-016-0596-1 (PMC4728774; doi:10.1186/s12862-016-0596-1)

Gnathostome TAB orthology

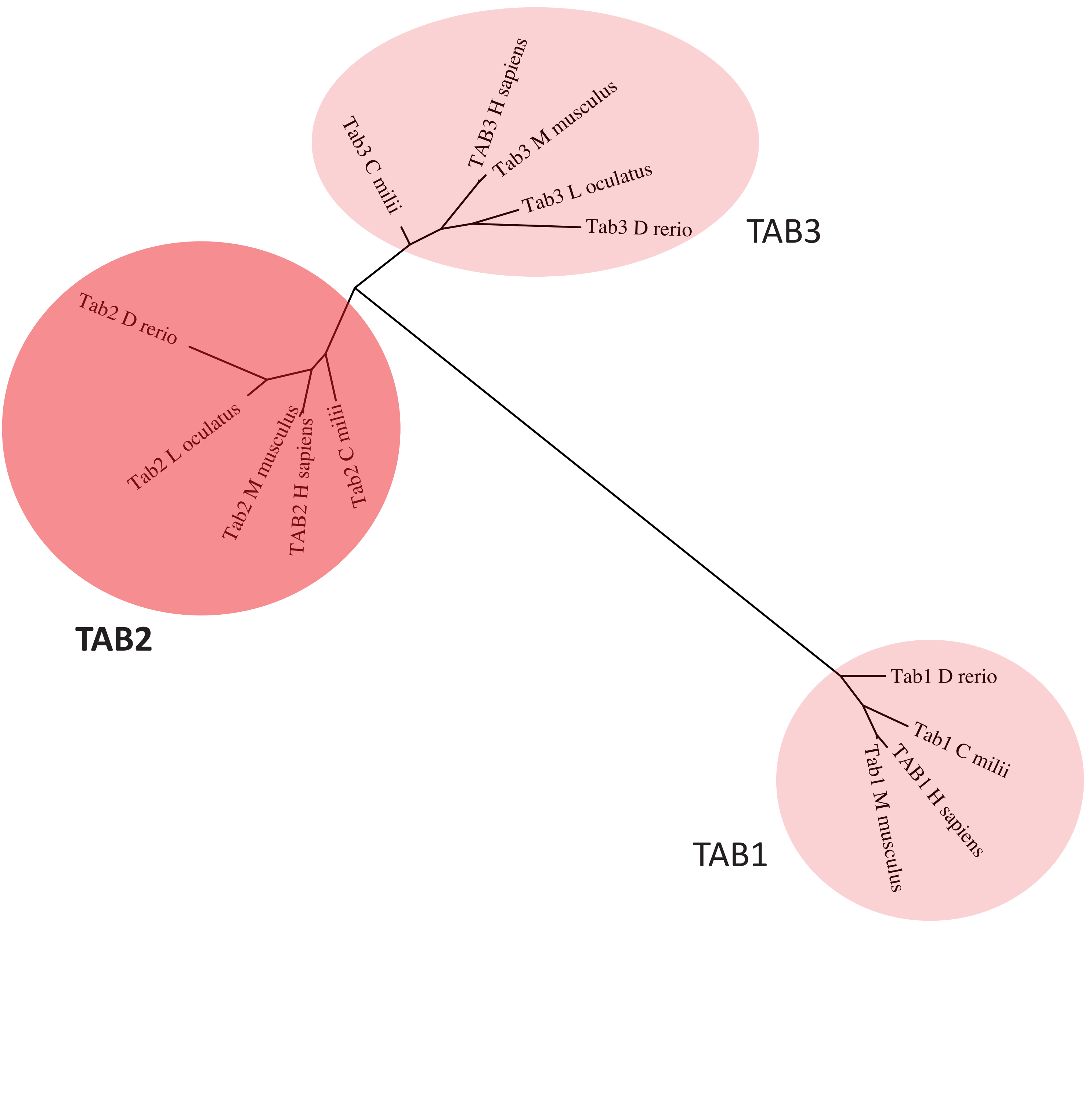

Gnathostome Nox orthology

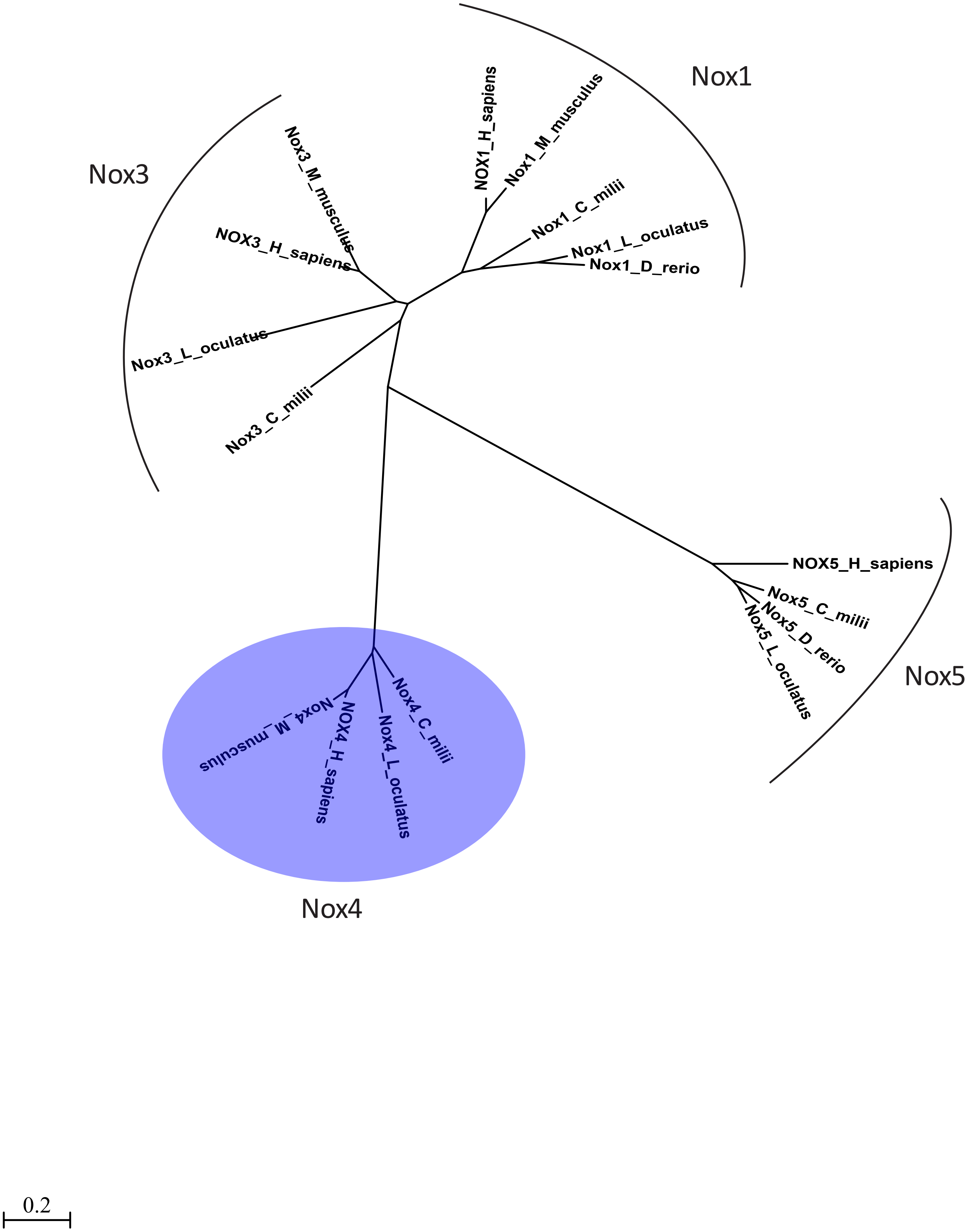

Gnathostome Fzd orthology

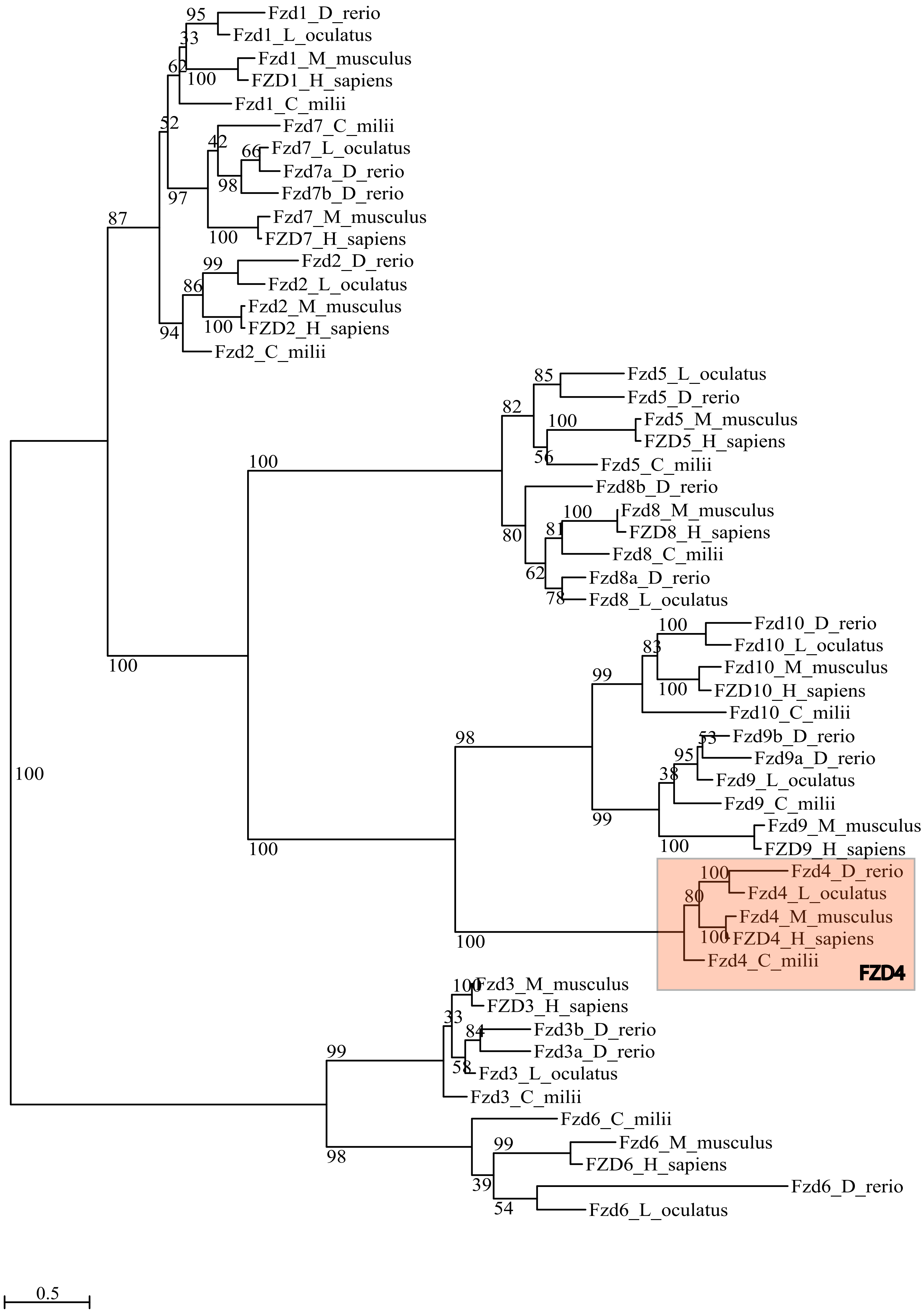

Gnathostome STXBP orthology

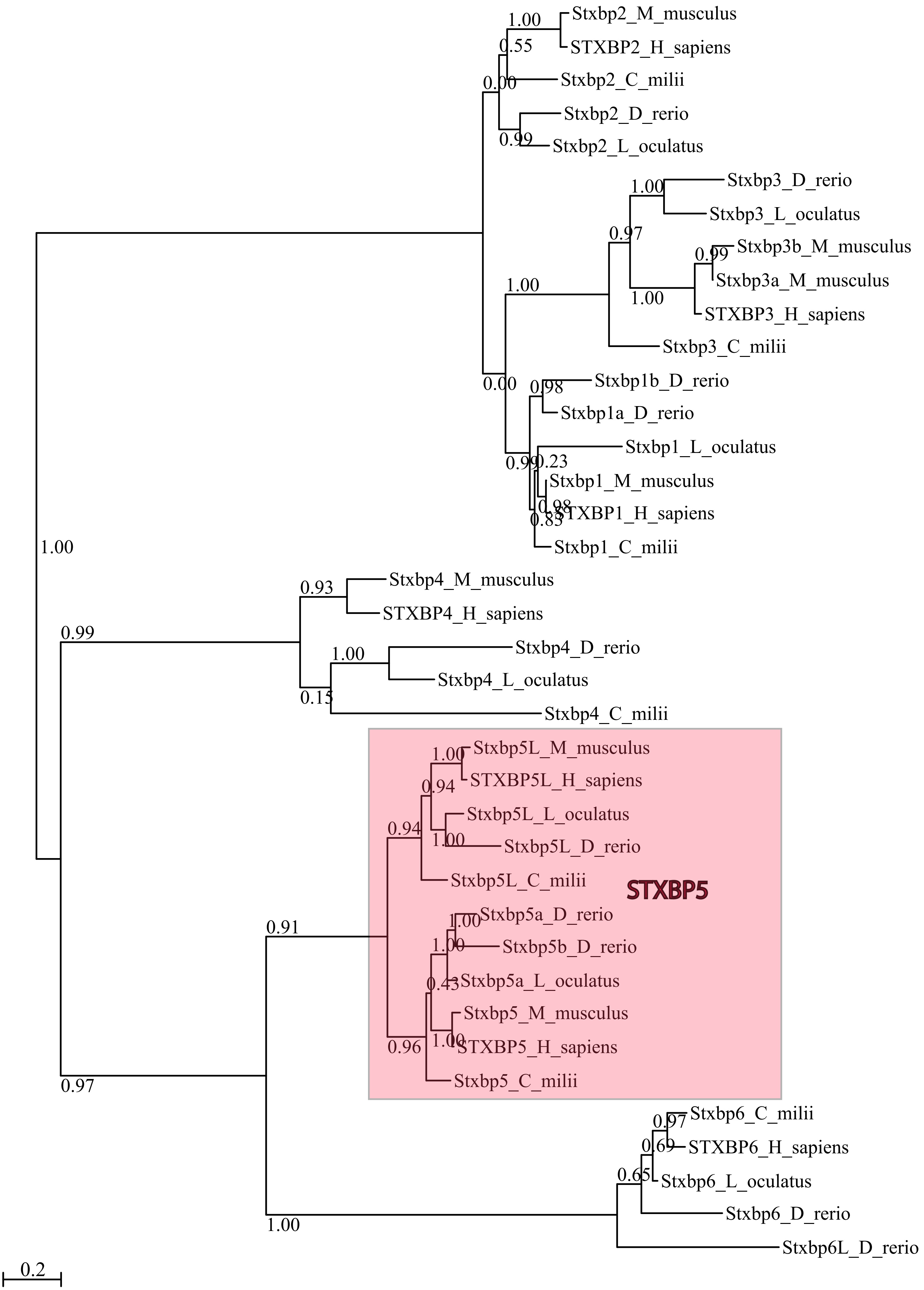

Supplement: Additional file 6: — Phylogenies of Tab, Nox, Fzd, Stxbp. (PDF 378 kb) [file 12862_2016_596_MOESM6_ESM.pdf]
