# Supplementary material for: Rab32 and Rab38 genes in chordate pigmentation: an evolutionary perspective
Source: BMC Evol Biol. 2016 Jan 27;16:26. doi: 10.1186/s12862-016-0596-1 (PMC4728774; doi:10.1186/s12862-016-0596-1)
Supplement: Additional file 8: — Additional syntenic studies. A. Human chromosome 11 and mouse chromosome 7: a focus on Rab38 gene loci with, respectively, a TRIM gene family expansion and an extraordinary clustering of olfactory genes: Vomeronasal 2 receptor (Vmn2r) and Olfactory Receptor (OR) in mouse; B Microsynteny of Rab32LO between echinoderms and cephalochordates. (PPTX 72.8 kb) [file 12862_2016_596_MOESM8_ESM.pptx]

## Slide 1
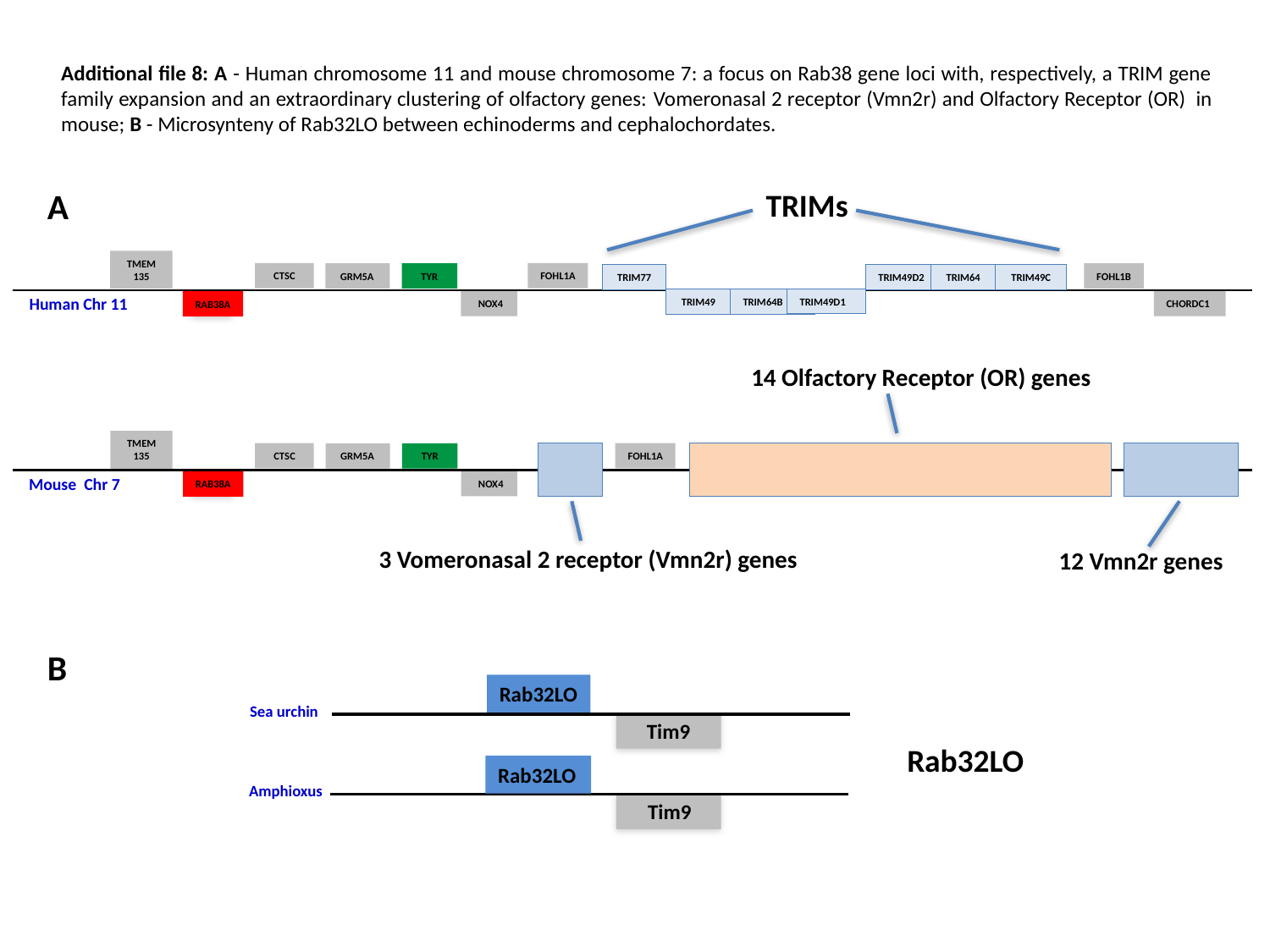

Additional file 8: A - Human chromosome 11 and mouse chromosome 7: a focus on Rab38 gene loci with, respectively, a TRIM gene family expansion and an extraordinary clustering of olfactory genes: Vomeronasal 2 receptor (Vmn2r) and Olfactory Receptor (OR) in mouse; B - Microsynteny of Rab32LO between echinoderms and cephalochordates.
A
TRIMs
TMEM
135
FOHL1A
CTSC
 GRM5A
TYR
FOHL1B
TRIM77
TRIM49D2
TRIM64
TRIM49C
Human Chr 11
TRIM49D1
TRIM64B
TRIM49
 NOX4
CHORDC1
RAB38A
14 Olfactory Receptor (OR) genes
TMEM
135
FOHL1A
CTSC
 GRM5A
TYR
Mouse Chr 7
 NOX4
RAB38A
3 Vomeronasal 2 receptor (Vmn2r) genes
12 Vmn2r genes
B
Rab32LO
Sea urchin
Tim9
Rab32LO
Rab32LO
Amphioxus
Tim9
