# Supplementary material for: Rab32 and Rab38 genes in chordate pigmentation: an evolutionary perspective
Source: BMC Evol Biol. 2016 Jan 27;16:26. doi: 10.1186/s12862-016-0596-1 (PMC4728774; doi:10.1186/s12862-016-0596-1)
Supplement: Additional file 9: — Gene expression profile during amphioxus development. Rab32/38 and Rab32LO gene expression profiles by real time RT-PCR during Branchiostoma lanceolatum development. Both genes have been normalized using the ribosomal protein L32 (RPL32) expression using the same cDNA template. (DOCX 41.4 kb) [file 12862_2016_596_MOESM9_ESM.docx]

**Additional file 9**: **Gene expression profile during amphioxus development**

Rab32/38 and Rab32LO gene expression profiles by real time RT-PCR during *Branchiostoma* *lanceolatum* development. Both genes have been normalized using the ribosomal protein L32 (RPL32) expression using the same cDNA template.

**Oligonucleotides for real time RT-PCR experiments**

| **Gene** | **Forward** | **Reverse** |
| --- | --- | --- |
| Rab32LO | TTCGACTTGTCAAGACCAAC | CTTGTCGAACGTCGAGTCAT |
| Rab32/38 | GGTTCGGAAACATGACAAGA | GTGACAAGATATCTGGCTGC |
| RPL32 | GGCTTCAAGAAATTCCTCGTC | GATGAGTTTCCTCTTGCGTGA |
